# Tested by Results

**Published:** 1890-08-09

**Authors:** 


### The Hospital

A Weekly Journal of

Science, Medicine, Nursing, and Philanthropy.

*For Hospitals, Asylums, Medical Practitioners, Students, Nurses, Families, Parishes, Congregations, and the Charitable Public.*

Vol. VIII.—No. 202.] [August 9, 1890.

### Tested by Results.

At the annual meeting of the British Medical Association held last week at Birmingham, Sir Walter Foster, in the course of an address on "The Public Aspects of Medicine," expressed the hope that at some not very distant day the State would awake to the value of the scientific services of medical men, and would "recognise in the trusty dispenser of a nation's charity, or the wise saviour of a city's health, servants of the State more worthy of its honours than the successful soldier or the astute diplomatist." Sir Walter, in his zeal for the honour of medicine, perhaps goes a little too far. But his fault, if there be one, is small in comparison with the conduct of the State, which does not go far enough in the direction indicated by him. The State, to some people, is a kind of dim abstraction to which they must yield a certain more or less willing obedience, but of which they themselves form no integral or responsible part. Most of the doctors who listened to Sir Walter Foster probably felt themselves very ill used by that same State, and did not recognise at all clearly that they constituted a very important part of it, and were, individually and collectively, considerably responsible for their own ill-usage. But when so much is freely admitted, it cannot be denied that the public authorities and the influential part of the population of this country are much less discriminating than they ought to be in the selection of particular citizens and classes of citizens for special State honours. In the common affairs of every-day life and business individual men are under the necessity of showing practical wisdom and judgment on pain of failure and degradation to the lowest social level. A shopkeeper, for example, learns to bestow much more real honour upon the farm labourer who steadily pays for his bacon and tea week by week than on the peer or soldier who eats the bacon and drinks the tea, but forgets to pay for them. Therein he exercises a rational and discriminating judgment which results in his financial progress and salvation. That material abstraction which we call the State ought, as far as possible, to act upon these very same principles. For everyone will admit that as the State is more powerful than the individual, so also it ought to be wiser, juster, and in every way more worthy of the admiring confidence of those over whom it exercises authority.

The question of the ill-usage of particular classes of citizens by State authorities may be approached either from the standpoint of the State, or from that of the ill-used classes. Sir Walter Foster approached it from the side of the doctors: we will look at it from the side of the powers that be. The State may for convenience sake be here regarded as the head of the body politic. One of the very first endowments demanded of a head is that it shall have understanding, measure, discrimination. It must be able to see clearly the specific and relative value of the different kinds of service that particular classes render the body politic, and it must know how to reward services according to their public and general worth. Moreover, it must have the wit to understand that the value of services is not a fixed value, but will vary from time to time with circumstances. Thus, for example, at certain periods of a State's history the soldier will be deserving of the highest honour, because he by his courage and resource may be the sole means of saving it from annihilation. Without him the State could not be. It is clear, then, from the point of view of the State, however it may be from that of plain sense and social philosophy, that the soldier must always be worthy of high honour, at least so long as men are such fighting animals as they are at present, and hitherto always have been in the world's history.

But if we admit the extreme value of the services rendered by the soldier when he saves the State as a State from extinction, what shall be said of the worth of that man's work who saves the lives of the particular individuals without whom the State could not so much as begin to be? A given State may be extinguished as a State, but the separate individuals may continue to live and even to be fairly happy. But when disease becomes rampant and triumphant, the existence, not only of the State, but of all its separate members, is put in danger. In a country like our own, thickly peopled, and where the people are under the necessity of possessing high powers of mind and body in order to survive in the daily struggle, it is clear that the doctor who keeps both disease and death at bay, and devises efficient measures for the increase of all kinds of human energy, not only deserves as high honours as the soldier, but is, indeed, the new soldier, more skilled, more subtle, and more farseeing, if not more valiant, than his predecessor, who fights the battles of the modern time. We grudge no honour to the heroes of past generations who have fought and carried victory in their breasts. But whatever triumphs the State has decreed to them must in all fairness now be given also to their successors who, in the laboratory, the museum, the hospital, and at the bedside face subtler and more dangerous foes, and drive them from the house and from the land.

Sir Walter Foster did not take up the strongest possible position when he chose the soldier and the diplomatist for contrast with the doctor. The merit of the soldier we have just considered. The diplomatist, if he be worthy of the name, can render the highest possible services to man, on the largest possible scale. He may avert the scourge of war; he may extend the dominion of peace. For such a diplomatist no honour the State may bestow can be too great. But in our time, so noble in some ways, so degenerate in others, the highest social and public honours are not reserved for the diplomatist and the soldier. They are given to the money maker. For this, as an integral part of the State, we blush and burn with shame. The soldier devotes his days and nights, his mind and body, his soul and life to the State. The diplomatist, too, lives for the service of his master. But for whom does the money maker toil and strive, and rack his brain and stretch his conscience until the last fibre cracks and yields, and integrity is known no more? For himself! For himself first, and last, and always! Yet it is for him that the exclusive social circle flings wide its arms; for him that the State adds honour to honour; upon him that beauty smiles and culture bestows its approving benediction; before him that all the world of wit, of learning, of fashion, and even of religion bends low and utters words of hypocritical reverence. The worship of the golden calf is the highest cult of the modern Englishman. The doctor, the life saver, the explorer of the mysteries of disease and death, the vanquisher of ten thousand of the subtlest and most dangerous foes: What is he in the general estimation? A poor pale creature who grubs in a laboratory, and propounds theories which it makes the head ache to understand.

The doctors have demanded worthier recognition of their work, but they have asked in vain. Why? Because they are not clamorous enough; because they have not the brazen face and the iron tongue of the man whose being is filled out and enlarged and magnified and made glorious by money. We are not looking at the subject from their point of view, but from that of the powerful yet powerless State. The reply of the State is that so many classes are more clamorous and more insistent than the doctors; and that the clamorous must be attended to first. This experience is as old as that of the unjust judge and the importunate widow, and indeed thousands of years older. To push, to elbow, to insist, to take no denial—that is the way of self-advancement; and that is an art that doctors have not yet learnt to practice with so much skill and resolution as some of their fellow men employ. The State, however, is bound to try to learn its own business. Although it may not possess all the power for good which ardent admirers of a paternal Government attribute to it, yet it may give such direction and impetus to national development as shall result in the triumph of justice, the progress of knowledge, and the crowning of the fittest with honours justly due. As medical men we have little need to care what the rulers of the people may think of us at any particular stage of our country's history. Time, which has raised the author of "Paradise Lost" to a just elevation, and crowned Shakespeare with a glorious immortality that has no parallel, will do complete, even if tardy, justice to those who have wrested from reluctant nature the innermost secrets of life and death. But as integral portions of a self-governing State, we are ashamed of our ignorant and foolish judgments, of our exaltation of the unworthy and our neglect of the meritorious, of the misdirection we have given to national progress, and of the poor and ignominious standard of public worth and virtue which we and our fellow countrymen have so universally set up.

